# Platelets/lymphocytes ratio and neutrophils/lymphocytes ratio in children with H. pylori associated gastritis: a canary in the mine?

**DOI:** 10.1038/s41390-025-04727-1

**Published:** 2026-01-13

**Authors:** Eman Elsaadany, Doaa El Amrousy, Shaima S. Qassem, Nesreen El Feil, Lamia M. Morad

**Affiliations:** 1 Department of Pediatrics, Faculty of Medicine, Tanta University, Tanta, Egypt; 2 Department of Clinical Pathology, Faculty of Medicine, Tanta University, Tanta, Egypt

## Abstract

**Background:**

Low-grade systemic inflammation and localized stomach inflammation are both brought on by *Helicobacter pylori* (H. pylori), which is a prevalent cause of chronic gastritis. The aim of this study was to assess the association between H. pylori positivity, severity, and both platelets/lymphocytes ratio (PLR) and neutrophils/lymphocytes (NLR) ratios in children.

**Methods:**

One hundred consecutive children who underwent gastroscopy for upper gastrointestinal tract symptoms were analysed retrospectively. They were classified according to identification of the H. pylori antigen in stool before endoscopy and confirmed by gastric endoscopic biopsy into H. pylori positive and negative groups. Their complete blood count was analysed, and PLR and NLR ratios were calculated.

**Results:**

H. pylori*-*positive group had significantly more prevalent erythema, nodularity, enlarged folds, and active chronic gastritis. Neutrophil count, NLR, and PLR were significantly higher in the H. pylori*-*positive group compared to the H. pylori*-*negative group, and this increase positively correlated with the severity of the infection.

**Conclusion:**

H. pylori-positive patients have considerably higher NLR and PLR. Moreover, PLR and NLR were good predictors for the presence and severity of H. pylori infection in children with upper gastrointestinal tract symptoms.

**Impact:**

- Low-grade systemic inflammation and localized stomach inflammation are both brought on by Helicobacter pylori (H. pylori), which is a prevalent cause of chronic gastritis. Recent adult studies reported that there is an association between H. pylori infection and both platelets/lymphocytes ratio (PLR) and neutrophils/lymphocytes (NLR) ratio.
- This study aimed to assess the association between H pylori positivity, severity, and both PLR and NLR ratios in children.
- We found that H. pylori-positive patients have considerably higher NLR and PLR. Moreover, PLR and NLR were good predictors for the presence and severity of H. infection in children with upper gastrointestinal tract symptoms.

## Introduction

In pediatric patients, persistent dyspeptic symptoms such as vomiting, nausea, and belly discomfort possibly indicate the presence of gastritis and gastropathies. *Helicobacter pylori* (H. pylori) is considered one of the most frequent causes of gastritis.^1^ H. pylori is a microaerophilic gram-negative bacterium that frequently resides on the luminal surface of the stomach epithelium.^2^ Around 30% of children suffer from this bacterial infection, which is more common in developing countries.^3,4^

The majority of children who have H. pylori infection are asymptomatic. However, H. pylori may gradually harm the stomach mucosa, which may result in a number of stomach disorders such as gastric cancer, peptic ulcers, chronic gastritis, and mucosal-associated lymphoma (MALT).^5^

Furthermore, H. pylori infection has the ability to trigger extra-gastric diseases such as idiopathic thrombocytopenic purpura, iron deficiency anemia (IDA), cardiovascular disorders, skin conditions, and immunological diseases because of the systemic subclinical inflammatory status, so diagnosis and early eradication are important.^1,6^

Localized stomach inflammation and mild systemic inflammation are both brought on by H. Pylori. Neutrophils and lymphocytes are drawn to the stomach by several chemotactic proteins produced there by H. pylori infection. Mucosal inflammation results from the secretion of several chemicals by neutrophils and mononuclear cells. Low-level inflammation in the stomach results from the infiltration of neutrophils, macrophages, and lymphocytes into the stomach mucosa, which is accompanied by a systemic humoral immune response through the activation of many cytokine signals. Neutrophils and lymphocytes are the main cell types that rise or decrease in the circulation in response to the physiological reaction of leukocytes to stress.^7^

Platelets/lymphocytes ratio (PLR), neutrophils/lymphocytes (NLR) ratio, erythrocyte sedimentation rate, and C-reactive protein were among the laboratory parameters that were evaluated in multiple studies for their feasibility in identifying the inflammation linked to this infection and differentiating between the sources of hematologic disorders.^8^ Of all these elements, NLR and PLR are the most crucial since neutrophils are an essential part of the immune system, and platelets participate in normal homeostasis as well as pathological states such as bleeding and thrombosis.^9^ This study aimed to determine if there is an association between PLR and NLR and H pylori positivity and severity in children.

## Patients and methods

In this retrospective study, we looked over the electronic medical records of all children with upper gastrointestinal (GIT) tract symptoms who underwent upper GIT endoscopy and were admitted to the Pediatric Gastroenterology and Hepatology Unit, Tanta University Hospital, between January 2021 and October 2023. This study was carried out according to the ethical guidelines of the Helsinki Declaration and its consequent amendments, with approval from Faculty of Medicine, Tanta University’s ethical committee (36264PR454/12/23). Being a retrospective study, the ethical committee put aside the requirement for informed consent.

### Inclusion criteria

Pediatric patients who underwent upper GIT endoscopy due to upper GIT symptoms recurrent abdominal pain, vomiting, dyspepsia, hematemesis or melena, were included.

### Exclusion criteria

Children who had H. pylori therapy within the last six months, children taking antibiotics, H_2_-receptor blockers, or proton pump inhibitors three months before to the study, children with chronic diseases, children taking long-term medications that could affect hematologic parameters and severity of gastritis as non-steroidal anti-inflammatory drugs (NSAID) and steroid, children with other infections, and children with parasitic infections were excluded from the study by routine stool analysis.

We recruited one hundred children and divided them into two groups based on whether they had a positive H. pylori infection or not:

Group I: 56 children who were positive for H. pylori infection

Group II: 44 children who were negative for H. pylori infection.

The subsequent information was extracted from the patients’ medical files: age, sex, family history, clinical presentation, and anthropometric measurements, including weight, height, and body mass index.

The diagnosis of H. pylori was made by the identification of the H. pylori antigen in the stool before endoscopy as a screening noninvasive test and confirmed by gastric endoscopic biopsy. Following upper GIT endoscopy (Pentax, 9.8 mm, EG-2990i, Japan), samples of tissue were acquired for assessment and histological identification of H. pylori infection in all recruited individuals.

To identify the H. pylori infection, at least five to six stomach biopsies were taken. According to NASPGHAN/ESPGHAN guidelines, a minimum of three biopsies should be collected from both the antrum and the corpus. In our cohort, two samples, at least, were collected from the corpus and two from the antrum for histopathological examination due to procedural and clinical considerations, including minimizing endoscopy duration and sedation exposure, reducing the risk of bleeding from inflamed mucosa, and adhering to institutional protocol limits on biopsy numbers. The updated Sydney approach classified H. pylori density, activity, chronic inflammation, and atrophy into four unique grades: none, mild, moderate, and severe.^10^

H. pylori activity was determined by neutrophil polymorph infiltration of the surface epithelium, pits, or lamina propria. The results were divided into four categories; none: indicates polymorphs that are hard to detect, mild: the pit and surface invasion was less than one-third, moderate: about 1/3 and 2/3 of the pit and surface had been invaded, and severe: the invasion of the pit and surface exceeded two thirds.^11,12^

The following criteria were used to evaluate chronic inflammation; grade 0 inflammation: indicates that there is no increase in lymphocytes or plasma cells, grade 1: indicates a slight rise in density (mild), grade 2: indicates a moderate rise in density (moderate), grade 3: indicates a severe rise in density (severe).

Loss of specific glands from the body or antrum was described as atrophy and could be classified as none, absent, mild, moderate, or severe.^11,12^

Laboratory investigations: complete blood count, which included absolute values of hematocrit (Htc), hemoglobin (Hb), red blood cell volume distribution width (RDW), mean corpuscular volume (MCV), platelet count, leukocyte count, and mean platelet volume (MPV), were performed at diagnosis during hospital admission.

Platelet-to-lymphocyte ratio (PLR) and neutrophil-to-lymphocyte ratio (NLR) were calculated. PLR and NLR values were calculated by dividing the absolute number of platelets and neutrophils by the absolute number of lymphocytes, respectively.

### Statistical analysis

The gathered data were arranged, tabulated, and statistically analyzed using Statistical Package for the Social Sciences (SPSS) software version 26 (IBM Crop, Armonk, NY).^13^ Quantitative data were presented in the form of mean and standard deviation (SD), while qualitative data were presented in the form of percentages and numbers. Comparing qualitative data in the two groups was performed using the Chi-square test (*χ*2). Comparing the means of the two groups was performed using the Student *t* test. The means of the severity grades were compared using the one-way analysis of variance (ANOVA) test. We evaluated the correlation between the variables using Spearman’s correlation coefficient. Regression analysis was performed to determine predictors for the presence and severity of H. pylori infection. Interpreting the results of tests of significance required using a significance threshold of *P* < 0.05.

## Results

One hundred recorded children underwent upper GIT endoscopy due to upper GIT symptoms, which were divided according to H. pylori infection into two groups (Fifty-six patients with positive H. pylori infection and forty-four patients with negative H. pylori infection). There were no significant differences between the two groups as regards age, sex, weight, height, GIT symptoms, or family history for H. pylori infection. However, BMI was significantly lower in H. pylori positive patients compared to the control group (Table 1).

**Table 1:** Demographic data, anthropometric measurements, and clinical presentation of studied groups.

Characteristics	H. pylori		*P*
	(+) *n* = 56	(−) *n* = 44	
Age (years)	9.4 ± 3.0	9.5 ± 2.9	0.959
Sex			
Male, *n* (%)	24 (42.9%)	20 (45.5%)	0.871
Female, *n* (%)	32 (56.1%)	24 (54.5%)	
Family history			0.334
Negative *n* (%)	14 (25%)	36 (81.8%)	
Positive *n* (%)	42 (75%)	8 (18.2%)	
Weight (kg)	27.6 ± 10.1	31.9 ± 10.0	0.316
Height (cm)	130.2 ± 17.2	130.3 ± 16.1	0.841
BMI (kg/m^2^)	15.8 ± 1.8	17.9 ± 2.0	0.006
Clinical presentation			
Hematemesis	18 (32.1%)	10 (22.7%)	0.248
Abdominal pain	52 (92.9%)	42 (95.5%)	0.987
Vomiting	13 (23.2%)	8 (18.2%)	0.444
Melena	14 (25%)	13 (29.5%)	0.334

*BMI* body mass index.

According to gross endoscopic findings, gastric erythema, nodularity, and enlarged folds were significantly more prevalent in H. pylori positive group. Moreover, active chronic gastritis was significantly higher in H. pylori-infected patients compared to non- infected patients (Table 2).

**Table 2:** Comparison between H. pylori-positive and negative groups regarding gross and histopathologic findings by endoscopy.

Characteristics	H. pylori		*P*
	(+) *n* = 56	(−) *n* = 44	
Gross picture			
Erythema	46 (82.1%)	25 (56.8%)	0.002
Friability	15 (26.8%)	9 (20.5%)	0.467
Enlarged folds	5 (8.9%)	0 (0.0%)	0.041
Flat erosions	5 (8.9%)	2 (4.5%)	0.400
Ulcer	4 (7.1%)	0 (0.0%)	0.079
Nodularity	36 (64.3%)	6 (13.6%)	0.001
Histopathology			
Inactive chronic gastritis			
Mild	0 (0.0%)	27 (61.4%)	
Moderate	11 (19.6%)	9 (20.5%)	
Severe	2 (3.5%)	1 (2.3%)	<0.001
Active chronic gastritis			
Mild	21 (37.5%)	4 (9.1%)	
Moderate	18 (32.1%)	3 (6.9%)	
Severe	4 (7.1%)	0 (0.0%)	

Regarding hematological results, no significant difference was noticed between the studied groups as regards Hb, MCV, RDW, total leukocytic count, platelet count, MPV, or lymphocyte counts. However, neutrophil counts, NLR, and PLR were significantly higher in H. pylori-positive group compared to H. pylori-negative group (Table 3).

**Table 3:** Hematological findings between H. pylori-positive and negative groups.

Characteristics	H. pylori		*P*
	(+) *n* = 56	(−) *n* = 44	
Hemoglobin	10.2 ± 1.4	10.7 ± 1.5	0.110
MCV	79.5 ± 11.0	81.1 ± 3.2	0.511
Platelets	257.3 ± 46.	267.8 ± 37.2	0.119
TLC	6652.8 ± 1101.1	6248.3 ± 619.5	0.140
Neutrophil	58.6 ± 10.9	50.6 ± 5.7	0.001
Lymphocyte	33.9 ± 6.7	35.6 ± 4.5	0.088
NLR	2.9 ± 0.6	1.7 ± 0.2	0.042
PLR	134.4 ± 22.4	114.5 ± 27.5	<0.001
MPV	9.2 ± 0.2	9.2 ± 0.2	0.446
RDW	12.0 ± 0.7	12.5 ± 0.8	0.727

*MCV* mean corpuscular volume, *TLC* total leukocytic count, *NLR* neutrophil leukocyte ratio, *PLR* platelets leukocyte ratio, *MPV* mean platelet volume, *RDW* red cell distribution width.

Total leukocytes, total neutrophils, NLR, and PLR demonstrated a considerable increase with increasing severity of H pylori gastritis, whereas lymphocytes demonstrated a significant decrease with increasing severity of gastritis in positive H. pylori infected patients (Table 4). There was a significant positive correlation between the severity of gastritis in H. pylori-infected patients and neutrophil count, NLR, and PLR. However, lymphocytic count showed a non-significant negative correlation with the severity of H. pylori (Table 5).

**Table 4:** Comparison of hematological findings by the severity of H. pylori gastritis.

Characteristics	H. pylori-positive gastritis			*P*
	Mild (*n* = 21)	Moderate (*n* = 18)	Severe (*n* = 4)	
Hemoglobin (g/dl)	10.9 ± 1.9	10.2 ± 1.8	9.5 ± 0.3	0.410
MCV (fL)	81.0 ± 3.4	81.3 ± 3.7	82.3 ± 5.0	0.761
Platelets	249.8 ± 10.5	232.9 ± 6.9	216.7 ± 11.3	0.062
TLC	5800.5 ± 445.1	6970.5 ± 690.3	8950.0 ± 86.6	<0.001
Neutrophil	50.7 ± 7.3	60.4 ± 7.2	76.7 ± 2.9	<0.001
Lymphocyte	34.7 ± 4.8	30.6 ± 5.8	15.0 ± 0.1	0.001
NLR	1.4 ± 0.3	2.1 ± 0.3	2.5 ± 0.7	0.010
PLR	104.9 ± 7.5	139.0 ± 22.6	154.0 ± 18.9	0.001
MPV	9.2 ± 0.2	9.2 ± 0.2	9.3 ± 0.4	0.795
RDW	12.4 ± 10.7	12.5 ± 0.8	12.5 ± 0.5	0.890

*MCV* mean corpuscular volume, *TLC* total leukocytic count, *NLR* neutrophil to lymphocyte ratio, *PLR* platelet to lymphocyte ratio, *MPV* mean platelet volume, *RDW* red cell distribution width.

**Table 5:** Correlation between severity of H. pylori gastritis and some hematological findings.

Parameters	Severity of H. pylori	
	*r*	*P*
NLR	0.395	0.014*
PLR	0.5480	<0.001*
Neutrophil	0.413	0.03*
Lymphocyte	–0.173	0.874

*NLR* neutrophil to lymphocyte ratio, *PLR* platelet to lymphocyte ratio.

^*^Significant results.

Table 6 showed that both PLR and NLR were good predictors for the presence and severity of H. pylori in children with dyspeptic GIT symptoms.

**Table 6:** Predictors for the presence and severity of H. pylori in the studied children.

Parameters	Presence of H. pylori			Severity of H. pylori		
	OR	*P*	95% CI	OR	*P*	95% CI
NLR	3.4	0.001	0.51-0.92	1.9	0.01	2.34–7.41
PLR	2.1	0.03	2.31-4.52	1.5	0.04	3.60–4.21
Neutrophil	1.1	0.872	0.90–5.87	1.3	0.691	0.54–10.34
lymphocyte	0.653	0.175	0.26–9.06	0.842	0.748	0.69–1.35
Platelet	0.453	0.861	0.82–1.50	0.596	0.401	0.99–7.71

*NLR* neutrophil to lymphocyte ratio, *PLR* platelet to lymphocyte ratio, *OR* odds ratio, *CI* confidence interval.

## Discussion

Peptic ulcer illness and chronic gastritis in both adults and children are believed to be significantly influenced by H. pylori infection, which is the most prevalent chronic bacterial infection in humans.^13–15^ This causes the stomach to become locally inflamed, and it triggers a humoral immune response in the body that causes neutrophils to proliferate and lymphocytes to decline.^7^ Previous studies have used white blood cells, platelets, neutrophils, lymphocytes, and MPV levels, as well as their ratios, as inflammatory biomarkers. NLR and PLR are two of the most significant inflammatory markers.^9^ This work was conducted to assess the association between both NLR and PLR with H. pylori infection and the severity of infection in children with upper GIT tract symptoms.

It has been proposed that H. pylori infection and growth retardation may be caused coincidentally by the same factors, such as low socioeconomic status. Moreover, H. pylori may cause dyspeptic symptoms and IDA, which decreases food intake and causes malnutrition that affects growth. In addition, H. pylori causes low-grade chronic gastric and systemic inflammation through the release of cytokines that can also affect the growth.^16,17^ Another theory for the possible effect of H. pylori on the growth is that the organism can affect the hormones that regulate appetite.^18^ Our study showed that the anthropometric measurements of the studied children were insignificantly different between the two studied groups, except for BMI, which was significantly lower in H. pylori positive group. Results of research looking at how H. pylori infection affects growth in children are noticeably contradictory; whereas some studies found a negative relationship,^16–19^ others did not.^20,21^ These contradictory results may be caused by different durations of infection, severity of infection, socioeconomic status, and different comorbidities in the included patients

In the current study, based on pathological stomach examination, active chronic gastritis was significantly more prevalent in children with positive H. pylori infection than in those with negative H. pylori infection. This showed that infection by H. pylori has a major effect on the intensity and activity of gastric inflammation. These results align with what other researchers have reported.^22,23^ Moreover, the gross upper endoscopic examination of the mucosa showed that gastric erythema, nodularity, and ulceration were more prevalent in patients who tested positive for H. pylori than in those who tested negative. This was consistent with the results of previous research.^22–24^

In our study, we noticed that HB, RDW, and MCV were reduced in patients with H. pylori infection than those with negative H. pylori infection but this decrease did not reach a significant difference. Several studies reported that H. pylori infection is associated with iron deficiency and vitamin B12 deficiency anemia and treating the infection provides a significant improvement in serum iron and vitamin B12 levels even without supplementation.^21,24,25^ Furthermore, the guidelines of both ESPGHAN and NASPGHAN in 2024 on management of H. pylori infection in children recommended that children with resistant IDA or failed optimal response to iron supplementation after exclusion of other causes of IDA should be tested for H. pylori infection.^26^

Regarding platelet count and MPV values, the two studied groups showed no significant difference, and this was in agreement with the results of a previous study.^25^ However, Agin et al.^27^ reported a slight correlation between the platelet levels and the presence of gastritis, despite the fact that neither the severity nor the presence of H. pylori infection was linked to MPV. Several studies have reported an association between H. pylori and immune thrombocytopenia in adult patients.^28,29^ There is little data on a possible association between H. pylori and thrombocytopenia in children.^30^ However, a previous meta-analysis reported that H. pylori eradication has a significant therapeutic effect in patients with ITP and suggested that H. pylori virulence factor, cytotoxin-associated gene A (CagA), stimulates the development of anti-CagA antibodies that cross-react with platelet surface antigens, resulting in thrombocytopenia.^31^

Our study reported that the total leukocytic count, as well as the lymphocytes, were comparable between H pylori-positive and H. pylori-negative children. However, the neutrophil count was significantly more elevated in the positive group than the negative group. These results were similar to those of the previous studies.^1,32^

NLR and PLR were found to be reliable inflammatory indicators for the diagnosis and staging of gastric cancer, whether used alone or in combination.^33^ Decreased lymphocyte count and increased neutrophil and platelet count were the causes of high values of NLR and PLR in cases of gastritis induced by H pylori infection.^34^

In a study conducted by Sasaran et al.,^25^ they found an insignificant increase in PLR and NLR values in H pylori-induced gastritis in children than those with H pylori-negative gastritis, but without a statistically significant difference. On the contrary, other researchers reported no relationship between NLR and MPV values and childhood H. pylori infection, severity classification, or pre- and post-treatment status.^35,36^ The difference in results may be related to the duration and severity of H. pylori infection, different age group, and socioeconomic status of the patients.

NLR and PLR in our study were significantly more elevated in the positive group than the negative group, and this elevation was significantly correlated with the severity of H. pylori infection. These were in agreement with Jafarzadeh et al.,^32^ who found significantly higher total leukocytic count, neutrophil count, and NLR values in adult patients with H. pylori-infected peptic ulcer compared to the control group. Similarly, Boyuk et al.^37^ found that PLR value in the adult H. pylori positive group increased significantly more than that in the H. pylori negative group, and the level of PLR was found to be proportional to H. pylori-associated symptoms.

It is currently unclear what precise mechanism causes patients with H. pylori infection to have elevated NLR. The elevated NLR in patients with H. pylori infection and its association with the severity of the pathology of the disease have been explained by several theories. H. pylori triggers inflammatory pathways that result in systemic and local gastric inflammation that elevates several inflammatory markers, including neutrophil, NLR, and PLR.^38^ Moreover, H. pylori produces heat shock protein that activates T cells and macrophages, which in turn produce several inflammatory cytokines such as interleukin-1 (IL-1), IL-6, IL-8, and tumor necrosis factor alpha.^38,39^ In addition, some H. pylori strains, such as CagA-positive H. pylori strains, induce larger levels of IL-8, which leads to more complex gastroduodenal disorders like atrophic gastritis, DU disease, and gastric cancer.^40,41^

NLR and PLR significantly increase with increasing severity of H. pylori infection and were positively correlated with the severity of the disease. Moreover, PLR and NLR were good predictors for the presence and severity of H. infection in children with dyspeptic symptoms. Based on this result, PLR and NLR might be easily obtainable parameters in the detection and prediction of severity of H. pylori infection.

We emphasize that Platelet/lymphocyte and Neutrophil/lymphocyte ratios are simple, cost-effective indices of systemic inflammation that may reflect disease severity in children with H. pylori gastritis. Although these ratios cannot replace diagnostic endoscopy, their accessibility makes them attractive adjunctive biomarkers for clinical decision making. Elevated ratios may help prioritize children for endoscopy in resource-limited countries, and could be used as supplementary indicators for monitoring inflammatory improvement after eradication therapy. Therefore, they can be used for follow-up and assessment of treatment response, particularly when confirmatory testing is delayed. However, future prospective pediatric studies are required to validate their diagnostic and prognostic utility.

Limitations of the study: This study has several limitations. First, the retrospective nature of the study. Second, we did not measure other inflammatory markers such as IL-1 and IL-8, and lack of follow-up after treatment. Third, it was conducted at a single center in a developing country with a higher prevalence of H. pylori infection. Fourth, our sample consisted exclusively of hospitalized patients, which may cause a severity-related selection bias. Lastly, several endoscopic features, such as mucosal edema or enlarged folds, are subjective and may vary between observers.

## Conclusion

NLR and PLR are thought to be recent, widely used, and affordable metrics for determining the level of inflammation in a variety of systemic illnesses. In our study, H. pylori-positive patients have considerably higher NLR and PLR. Moreover, gastric histopathological findings correlated significantly with NLR and PLR. PLR and NLR were good predictors for the presence and severity of H. pylori infection in children with upper GIT tract symptoms.

## Data Availability

Upon reasonable request, the corresponding author can supply the datasets utilized and/or analyzed for this research.
